# Mutation analysis and clinical characterization of Iranian patients with mucopolysaccharidosis type I

**DOI:** 10.1002/jcla.22963

**Published:** 2019-08-06

**Authors:** Mohammad Taghikhani, Shohreh Khatami, Mohammad Abdi, Mohammad Said Hakhamaneshi, Mohammad Reza Alaei, Daniel Zamanfar, Rahim Vakili

**Affiliations:** ^1^ Department of Clinical Biochemistry, Faculty of Medical Sciences Tarbiat Modares University Tehran Iran; ^2^ Department of Biochemistry Pasteur Institute of Iran Tehran Iran; ^3^ Cellular and Molecular Research Center Research Institute for Health Development Kurdistan University of Medical Sciences Sanandaj Iran; ^4^ Department of Clinical Biochemistry, Faculty of Medicine Kurdistan University of Medical Sciences Sanandaj Iran; ^5^ Department of Pediatric, Faculty of Medicine Shahid Beheshti University of Medical Sciences Tehran Iran; ^6^ Department of Pediatric, Faculty of Medicine Mazandaran University of Medical Sciences Sari Iran; ^7^ Department of Pediatrics, Imam Reza Hospital Mashhad University of Medical Sciences Mashhad Iran

**Keywords:** IDUA, Iranian population, mucopolysaccharidosis type I, mutation analysis

## Abstract

**Background:**

Mucopolysaccharidosis type I (MPSI) is a rare autosomal recessive disorder caused by a deficiency of α‐l‐iduronidase (IDUA) encoded by the *IDUA* gene. We examined the mutation spectrum of the *IDUA* gene to explain the clinical, biochemical, and molecular features in 21 Iranian patients with MPSI.

**Methods:**

Sanger sequencing was used to measure the *IDUA* gene sequence in the coding region and exon‐intron boundaries. We recorded the clinical findings of studied patients at the first diagnosis of disease and then during the treatment and follow‐up.

**Results:**

Five different missense disease‐causing mutations were determined in our patient groups, indicating 90.48% of detection rate. The most widespread mutation was the p.Y109H, occurring in 15.625% of all alleles, which was reported for the first time in our study. Other frequent mutations were as follows: p.Ser157Pro (12.5%), p.Gly84Arg (12.5%), p.Asp257His (9.375%), and p.Asp301Glu (9.375%). Three ones of them were new missense mutations: p.Ser157Pro, p.Asp257His, and p.Asp301Glu.

**Discussion:**

The results of this study explain the different spectrum of *IDUA* gene mutations in our patients with MPSI. We introduced here 32 different variants including four new variants: p.Y109H (15.625%), p.S157P (12.5%), p.D257H (9.375%), and p.D301E (9.375%). In this series, there was no relationship between the happening of clinical features and genotype variations and biochemical findings.

## 1 INTRODUCTION

Mucopolysaccharidosis type I (MPSI; MIM 252800) is an autosomal recessive disorder caused by the deficiency of α‐l‐iduronidase (IDUA; EC 3.2.1.76). The main role of IDUA is to catalyze the lysosomal degradation of two main glycosaminoglycans (GAGs) including heparan sulfate (HS) and dermatan sulfate (DS). The defect of IDUA enzyme is caused by mutations in the *IDUA* gene (NCBI Reference Sequence: NG_008103.1). Mucopolysaccharidosis type I is provided with different phenotypes and classified according to the severity of symptoms ranging from severe Hurler syndrome to restively mild Scheie syndrome.^1^ The progressive accumulation of DS and HS in lysosomes results in the severe decline of cells, tissues, and organs.^1^ Among the other approaches, enzyme replacement therapy with Laronidase (recombinant human IDUA; Aldurazyme (Genzyme Corporation) is mainly used to treat MPSI in United States, Europe, and many other countries.^2^ MPSI is considered to be one of the most commonly lysosomal storage disorders in different populations with a mean incidence of 1:100 000‐1:150 000 newborns.^3^, ^4^

The *IDUA* gene is located on chromosome 4p16.3 and covers nearly 19 kb. It includes 14 exons and encodes a 653 amino acid precursor protein.^5^, ^6^ Since the identification of the first MPSI allele, at least 201 different types of mutations and numerous nonpathogenic sequence variants in *IDUA* gene have been determined in various regions of the locus with the most being missense/nonsense mutations (HGMD® Professional release 2017.1; http://www.hgmd.cf.ac.uk/). The IDUA mutations, p.Trp402ter (rs121965019) and p.Pro533Arg (rs121965021), have been already reported to account for more than 50% of MPSI alleles in most populations.^7^ The influence of mutations on IDUA enzyme activity has broad variability, and thus, IDUA is provided in different forms; however, there is not a significant relationship between the *IDUA* genotypes and various phenotypes that are observed in MPSI. In addition, there is no proved significance relationship in studies published elsewhere between residual l‐iduronidase levels and MPSI phenotype. In the same way, there is also no clear genotype‐phenotype relationship for this disease. Therefore, the similar residual enzyme activity values can be obtained for described three phenotypes.^8^ Molecular characterization of the genetic mutational spectrum and polymorphism distribution may help in MPSI diagnosis and primary prediction of disease severity, providing accurate carrier detection and informative genetic counseling, which is important considering the clinical outcome and choice of treatment options.

It is seriously important to always determine and update the genotypic information of a population for a specific disease. In Iran, there is an insufficiency of studies based on a limited number of patients, and therefore, its application was confined to the needs at that time. Therefore, this study was aimed at measuring the mutation variety, compare it to other studies from neighbor regions and finally provide a study that indicates the current Iranian population.

## 2 MATERIAL AND METHODS

### 2.1 Subjects

Twenty‐one patients with MPSI who submitted to specialized pediatric clinics for Laronidase replacement therapy in different regions of Iran were enrolled in this study. All patients included in this study were determined at the outpatient clinic by a pediatrician for clinical features of MPSI. Cognitive ability was measured through clinical observation. A structured parental questionnaire was used to determine the information on pregnancy, first clinical signs, mental and motor milestones, behavioral problems, sleeping problems, and medical history. Typical facial appearance and other clinical findings such as macroglossia indicated MPSI. Tarbiat Modares University and Pasteur Institute of Iran research ethics committee approved this study that was conducted according to the Declaration of Helsinki for Medical Research Involving Human Patients. Written informed consents were obtained from the parents for molecular analysis and participate in the study. All patients were already diagnosed based on clinical presentation, quantitation and electrophoresis of urinary glycosaminoglycans, and finally by determining the low or nondetectable IDUA activity using a fluorometric measurement of enzyme activity.

### 2.2 Biochemical analysis

Quantitative measurement of urinary GAGs: Urinary GAGs was measured calorimetrically according to the reaction of DMB with urinary mucopolysaccharides according to our previous study, and results were expressed in mg/g creatinine.^9^

Fluorometric measurement of IDUA in dried blood spots (DBS): IDUA activity in DBS samples was measured according to our previously standardized method.^8^ Results were expressed as µmol/spot incubation time, and the low range results were confirmed by IDUA leukocyte assay.

### 2.3 Molecular study

Genomic DNA was extracted from venous blood using commercial kit of SinaClon Co. Multiple pairs of primers were used to reinforce 10 DNA fragments covering 14 exons and exon‐intron boundaries of the *IDUA* gene. Primers used to amplify the genomic sequences were designed according to the sequence NG_008103. Table 1 shows the primers, PCR product fragments, and annealing temperature for each pairs of primer. Direct DNA fragments sequencing was performed with an ABI‐3730XL capillary machine by BioNeer Inc according to Sanger sequencing technique. Patients’ genomic sequences are performed compared with the reference sequence by the CLC software (CLCbio). Finally, we compared our results with the published reference sequence. We also sequenced thirty healthy subjects to confirm whether the novel DNA alterations are causative mutations.

**Table 1:** Primer sequence and reaction condition

Product name	Primer sequence	Annealing temperature	Product size (bp)
Exon 1	F 5 > 3: GCGTTCTTCTGAGCGCTTT	59°C	576
	R 5 > 3: GAGGACCCACCCACAAACA		
Exon 2	F 5 > 3: TTTTATTAGTCACTGAACGCACG	60°C	333
	R 5 > 3: CCTCCCATCTGTGCCTCTGT		
Exon 3_4	F 5 > 3: CAGCCTGGAGCATGGAG	58°C	511
	R 5 > 3: GCGTGATAGGGGTGCAAC		
Exon 5_6	F 5 > 3: TCACCTTGCACCCTCCCTCC	62°C	536
	R 5 > 3: TGAGGGCGCAGAACACCG		
Exon 7	F 5 > 3: TGCGGCTGGACTACATCTC	59°C	442
	R 5 > 3: AGGTTCTGATGCTGCGC		
Exon 8	F 5 > 3: CCACCTTCCTCCCGAGAC	59°C	387
	R 5 > 3: GCTGGAGGAAGTGCGCT		
Exon 9	F 5 > 3: TCCTTCACCAAGGGGAGG	58°C	400
	R 5 > 3: CTGACACTCAGGCCTCGG		
Exon 10	F 5 > 3: GGTGACCCTGCGGCTG	60°C	421
	R 5 > 3: TCCTCAGGGTTCTCCAGG		
Exon 11_12	F 5 > 3: GTGTGGGTGGGAGGTGGA	58°C	459
	R 5 > 3: CATGGGTGAAGGGGTCG		
Exon 13_14	F 5 > 3: GCCTGCTCCCACCTTTGA	60°C	592
	R 5 > 3: AGGGGGCGTTGGTACCA		

### 2.4 Model building

**Figure 1:**
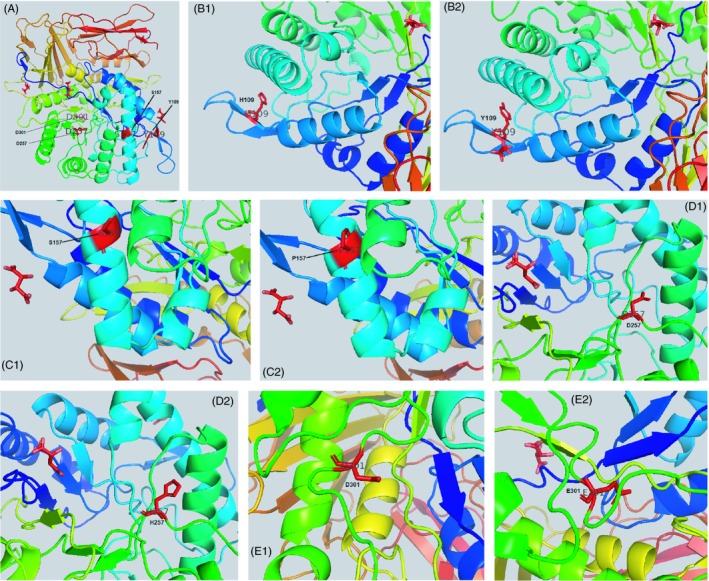
Modeling the three‐dimensional structure of the IDUA base on PDB 3W81: The wild‐type amino acids at position 109, 157, 257, and 301 of IDUA protein are illustrated (A). A close‐up view of nonmutated and mutated amino acid: p.Y109H (B), p.S157P (C), p.D257H (D), and p.D301E (E)

We have been used the three‐dimensional structure of human IDUA (PDB code 3W81) as a model to conduct the mutational studies. PyMol molecular virtualization software (The PyMOL Molecular Graphics System, Version 2.3 Schrödinger, LLC.) was used to exchange the site‐specific residue substitution (Figure 1).

## 3 RESULTS

The studied population included 21 unrelated MPSI patients belonged to the different regions of Iran which were admitted in specified hospitals in three provinces: Tehran, Mazandaran, and Khorasan. Patients included thirteen cases treated with Laronidase replacement therapy and eight new case patients. Patient group included 11 males and 10 females with the age range of 1.5‐13 years old. The percentage of consanguineous marriage was 71.43%, and all patients were born at term. Moreover, four cases of twenty‐one patients were born with a family history of other genetic disorders in other relatives. Three patients (P13, P16, and P18) were eutrophic, and three were macrosomic (P9, P10, and P14) (BW > 4 kg at term). Fifteen patients showed the facial dysmorphic features. There is macrocrania in nine patients (42.86%), macroglossia in five patients (23.81%), and an organomegaly in 18 patients (85.71%). There is umbilical hernia in seventeen patients, and there is a delayed growth in 18 out of 21 documented patients. All these patients had low height. Seven patients (P1, P4, P5, P6, P11, P16, and P21) were presented with a typical Hurler symptomatology. Ten of 21 patients were presented with HS form with mild mental retardation and four with mild S form of MPS I without mental retardation. Table 2 illustrates the main phenotypic symptoms and other features of studied subjects.

**Table 2:** Clinical characteristics of the studied subjects

Patients	Age (y)	Sex	Consanguinity	Diagnostic age (y)	Weight (kg)	Height (cm)	Mental retardation	Facial dysmorphic features	Multiple dysostosis	Joint stiffness	HM/HSM	Hernia	Chronic rhinorrhea	Deafness	Corneal opacity	Cardiac manifestations	Hydrocephalus
1	5.2	F	−	0.6	15	75	−	−	+	+	+	−	−	−	−	MS	−
2	4.2	M	+	0.9	11	65	−	−	±	+	±	−	−	−	−	−	−
3	1.5	M	+	0.2	7.5	72	−	−	−	−	−	−	+	−	−	−	−
4	7.2	F	+	0.3	23	112	−	−	+	+	+	−	−	−	−	−	−
5	10.5	F	+	1.2	10.5	87	+	+	+	+	+	+	+	+	−	MS/AI	+
6	13.0	M	+	0.9	15	102	+	+	+	+	+	+	+	+	−	−	+
7	12.9	F	+	0.7	14.2	97	+	+	+	+	+	+	+	+	−	−	+
8	1.3	F	+	0.7	7.0	70	−	−	−	−	−	+	+	−	−	−	−
9	1.2	F	+	0.7	6.8	69	−	−	−	−	+	+	+	−	−	−	−
10	7.8	M	−	0.6	9.0	74	+	+	+	+	+	+	+	+	−	−	+
11	6.2	F	−	0.5	14.5	100	+	+	+	+	+	+	+	+	+	−	−
12	10.1	F	+	0.9	26	108	−	+	−	−	+	+	−	−	−	−	−
13	1.6	M	−	0.8	7.2	71	+	+	−	−	+	+	+	−	−	−	−
14	8.5	M	+	0.7	9.5	73	−	+	−	−	+	+	−	−	−	−	−
15	4.2	M	−	0.7	10.1	60	+	+	−	−	+	+	−	−	−	−	−
16	5.5	M	+	0.6	11.0	66	+	+	−	−	+	+	−	−	−	−	−
17	3.0	M	+	0.9	9.0	66	+	+	−	−	+	+	−	−	−	−	−
18	9.2	M	+	1.3	20	89	+	+	−	−	+	+	−	−	−	−	−
19	2.2	M	+	0.5	8.0	7.6	+	+	−	−	+	+	−	−	−	−	−
20	12.5	F	−	1.3	23	80	−	+	−	−	−	+	−	−	−	−	−
21	2.8	F	+	0.8	7.5	80	+	+	+	+	+	+	+	+	+	MS	+

Abbreviations: −, absent; +, present; AI, aortic insufficiency; F, female; HM, hepatomegaly; HSM, hepatosplenomegaly; M, male; MS, mitral stenosis.

**Figure 2:**
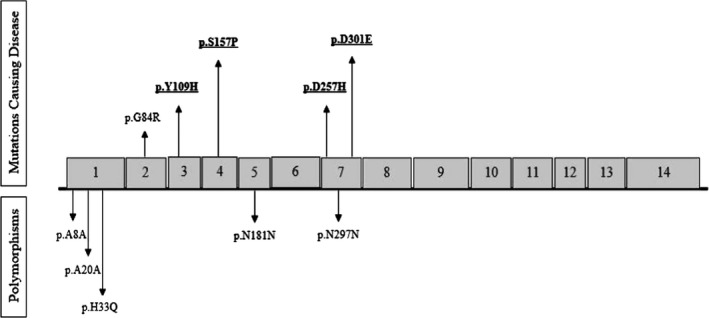
Spectrum of variants found in the present study: the numbered boxes represent each exon, and the vertical bars show the positions of the respective variants. The novel variants found out in this study are underlined

We have analyzed the genomic DNA of 14 exons of *IDUA* gene including intron‐exon regions by direct sequencing of PCR products. Our results identified 32 *IDUA* variant alleles from 21 patients, including three variant types: four new variants (in 15 alleles) and two previously described single nucleotide substitutions as missense (in 10 alleles), and four as silent (synonymous variants in seven alleles) (Figure 2). The main results of the study were as follows: NM_00203.4: g.18641T > C‐p.Tyr109His mutation was the most common variation in our patients observed in 26.32% of them. In short, there was the p.Tyr109His allele in five patients as homozygote genotype (include three male and two females). This mutation is first common new sequence variation caused by T to C change at position 18,641 of the *IDUA* gene (exon 3), resulting in a tyrosine to histidine substitution at residue 109 of the protein. Each NM_00203.4: g.18969 T > C‐p.Ser157Pro and NM_00203.4: g.5904 G > C‐p.Gly84Arg were identified in four alleles (21.05%). We observed the p.Ser157Pro new mutation as homozygote in four male patients. Our results also indicated p.Gly84Arg in four patients includes two males and two females, although this mutation is already described. The NM_00203.4: g.19882 G > C‐p.Asp257His and also NM_00203.4: g.20096 G > C‐p. Asp301Glu happen as third most widespread Iranian mutations in three (15.79%) alleles. p.Asp301Glu and p.Asp257His missense mutations had not also been previously described. In addition, we also identified one benign missense and four silent variants in studied patients. Previously described and new variants, nucleotide alteration, amino acid changes, and their frequencies of our study are listed in Table 3. For new alteration of IDUA, Polyphen2 was used to perform in silico analysis and indicated that the p.Tyr109His, p.Asp301Glu, p.Ser157Pro, and p.Asp257His variants as pathogenic according to the ACMG guidelines, while using this tool, p.Ser157Pro is realized to be a likely pathogenic variant. Table 4 describes all outputs and scores. Moreover, we identified five benign variants, including A8A, A20A, H33Q, N181N, and N297N.

**Table 3:** Mutation spectrum in the Iranian population, allele frequencies and mutation characteristics

Mutation name	Systemic name	Allele frequency	Relative frequency (%)	Mutation type	*IDUA* gene region	Reference
p.Y109H	c.413T > C	5/32	15.625	Missense	g.18641T > C	Novel
p.S157P	c.557T > C	4/32	12.5	Missense	g.18969 T > C	Novel
p.G84R	c.338G > C	4/32	12.5	Missense	g.5904 G > C	16
p.D257H	c.877G > C	3/32	9.375	Missense	g.19882 G > C	Novel
p.D301E	c.991G > C	3/32	9.375	Missense	g.20096 G > C	Novel
p.H33Q	c.187T > G	6/32	18.75	Missense	g. 5187T > G	16
p.N181N	c.631T > C	1/32	3.125	Silent	g.19521T > C	16
p.N297N	c.979C > T	3/32	9.375	Silent	g.20084 C > T	16
p.A8A	c.112C > A	1/32	3.125	Silent	g. 5112C > A	16
p.A20A	c.148G > A	2/32	6.25	Silent	g.5148G > A	16

**Table 4:** In silico analysis of novel mutations using PolyPhen2 software, calculated HumDiv, and HumVar scores

Mutation name	cDNA	In silico analysis		Classification
		HumVar score	HumDiv score	
p.Y109H	c.413T > C	0.989	1.0	Probably damaging
p.S157P	c.557T > C	0.772	0.989	Possibly damaging
p.D257H	c.877G > C	0.998	1.0	Probably damaging
p.D301E	c.991G > C	1.0	1.0	Probably damaging

There was the lowest activity in patient 14. There was the highest urinary GAG exertion in patient 5. There was no significant relationship between genotype and related phenotype in the studied subject (data not shown). Table 5 shows the mutation‐caused disease and biochemical findings at the time of diagnosis in studied subjects.

**Table 5:** Spectrum of genotypes, their frequencies, genotype‐phenotype correlations, and biochemical findings in studied subjects

Number of patients	Genotype	Clinical phenotype	Qualitative urinary GAG	Quantitative urinary GAG (mg/g_Cr_)	IDUA activity (μmol/spot.20h)
P1	p.D257A	H	+	11.14	1.02
P2	p.S157P	HS	++	14.22	7.06
P3	p.Y109H	HS	+	10.80	0.76
P4	p.D257A	H	++	13.27	1.27
P5	p.D257G	H	++	14.80	1.69
P6	p.D301E	H	+	11.54	1.3
P7	p.G84R	HS	+++	10.11	0.86
P8	p.G84R	HS	+	6.7	1.06
P9	p.Y109H	HS	+	7.21	0.97
P10	p.G84R	HS	±	7.8	2.08
P11	p.D301E	H	+	7.82	1.04
P12	p.Y109H	HS	+	7.4	1.01
P13	p.S157P	S	+	7.4	0.75
P14	p.Y109H	HS	±	6.4	0.6
P15	p.G84R	HS	++++	7.42	0.23
P16	p.D301E	H	++	8.5	0.89
P17	p.Y109H	HS	+	7.8	0.76
P18	p.S157P	S	±	7.7	1.67
P19	p.S157P	S	±	8.4	3.72
P20	–	S	+	7.6	2.3
P21	–	H	+++	10.5	0.98

Abbreviations: H, Hurler; HS, Hurler‐Scheie; S, Scheie.

## 4 DISCUSSION

In this study, the clinical features, biochemical, and molecular profiling of *IDUA* gene have been analyzed in a series of MPSI population in the Iranian population.

The detected mutations in our population were very different from other recent studies where sequencing analysis of the *IDUA* gene was conducted.^10^, ^11^, ^12^, ^13^, ^14^, ^15^ Despite sequencing analysis of all 14 exons and exon‐intron boundaries of the *IDUA* gene, genotypes of two patients are still unknown. It is possibly explained in such a way that the disease‐causing mutations can be placed in the promoter region, at the polyadenylation site, or in deep intronic regions. It seems that one of the next‐generation sequencing methods which are becoming more available is a competent method to determine the mutations in these regions.

Recently, Yassaee VR and colleague introduce two new IDUA variants in two unrelated MPSI patients from Iran including c.523T > C (p. W175R) and c.612_615dup (p. S206Lfs*194). Their study also indicated that substitution of Arg with Trp at position 175 may change the active site structure and decrease IDUA activity, while the later frameshift variant disrupt the IDUA protein fields and result in severe phenotype of MPSI. Those variants were identified by direct sequencing and both of them indicated low enzyme activity of IDUA, although other clinical and laboratory findings seemed to be different in each patient.^16^ In another study, Atçeken et al^10^ analyzed 15 patients with MPSI from Turkey. They used direct sequencing to determine the disease‐caused mutations in *IDUA* gene and identify nine new different pathologic variants (c.494‐1G > A, c.793‐6C > G, c.793‐5C > A, p.M1L, p.Y64X, p.A327P, p.W402X, p.P533L, and p.R628X). In addition, Fahiminiya S and colleague studied consanguineous families in Qatar and indicated a SNP‐causing Hurler syndrome in *IDUA* gene using whole exome sequencing.^17^

Our data indicated a range of five different mutations; four of them have not previously been explained in other patients with MPSI. The p.Tyr109His mutation was the most widespread variation in our patients and report 15.625% of all alleles. Our study is the first report that identified this mutation. The tyrosine, an aromatic amino acid, was replaced with a positive charge amino acid, histidine. Moreover, this change arises from near Asn110 which is an important glycosylation site of IDUA protein. Close position of these two positive charge amino acids can significantly change the physicochemical properties and seriously reduce the IDUA enzyme activity.

The second widespread mutation (12.5% frequency rate in detected alleles) in Iranian patients with MPSI was p.Ser157Pro. In addition to the location of this substitution, the most important fact is to change the polar amino acid serine to proline which has a secondary amine group and distinctive cyclic structure. The exceptional conformational rigidity of proline has an effect on the secondary structure of IDUA compared with wild‐type Ser form.

p.Asp257His (9.375%) was the other new mutation in our studied population. Like the previous mutations, here, this amino acid is placed at the active site of IDUA enzyme and substitution of negative charge aspartic acid with a positive charge histidine might cause to disturb the active site of the mature protein.

The p.Asp301Glu mutation was also indicated for the first time in our study and detected in 9.375% of identified alleles. Our study indicates for the first time the substitution of aspartic acid 301 with glutamic acid in IDUA protein. This replacement is located in the active site of IDUA enzyme and may affect the iduronate‐enzyme interaction.

Variation in glycine 84 of *IDUA* gene to arginine was previously described in some populations. This substitution occurs near to the active site of IDUA, and in addition to gain of charge, it causes loss of main chain flexibility and also solvent available surface. We identified this mutation in 12.5% of our studied subjects. This is a rare mutation and highly effects on solvent available residues.^18^, ^19^

The HumVar score for the new substitutions was close to 1 (except for p.Ser157Pro that is 0.772), thus predicted the severe pathological effect of this mutation (Table 3). Moreover, we sequenced exons 3, 4, and 7 in at least 75 alleles of healthy persons and none of the four new variants was detected. However, in vitro analysis of the mutated IDUA enzymes is the most reliable way to confirm our predictions.

Unfortunately, we were not able to measure the genetic features in parents to prove the autosomal recessive pattern. In this series, there was no relationship between the clinical severities and either the urinary GAG concentration or the residual enzyme activity, and therefore, it explains that biochemical analysis was not significant to determine the clinical types of MPSI (Table 5). This issue may be explained by the effect of other genetic and epigenetic factors on the clinical variability, particularly in patients with late‐onset disease. Therefore, the large variation in the mutational spectrum of the *IDUA* gene is possibly considered as a main cause of the clinical heterogeneity of MPSI. Many works have been done to search for genotype‐phenotype correlation in patients with MPSI.^19^, ^20^ Ghosh et al^19^ analyzed the genotype‐phenotype in a cohort of 291 individuals with MPSI. They assumed that a severe phenotype would be specified in the presence of two severe variants. Their results indicated that nonsense and other truncating mutations are related to a severe phenotype. Despite a genotype‐phenotype association for certain missense mutations, there was no relationship between other missense variants and observed phenotype. Moreover, a few studied variants indicated heterogeneous phenotypes. Totally, there was a genotype‐phenotype relationships for most MPSI patients in UK based on Ghosh and colleague results. Moreover, Ou et al^20^ explained that in silico analysis of IDUA variants determines the severity of MPSI disease. There were several possible variants with phenotype correlation. They also created a step‐by‐step guideline to determine MPSI phenotype using bioinformatics tools.

There were the following inconsistencies in genotype‐phenotype correlations. In four cases, mutation p.Ser157Pro was involved. One homozygote for the p.Ser157Pro mutation was classified as HS, and three homozygous patients were classified as S. However, here it should be noted that another mutation rather than 14 exons and exon‐intron boundaries of the *IDUA* gene could be present, but undetected in our study. This mutation possibly might determine the metabolic phenotype.

Two patients with unknown genotype showed the most interesting contradiction in our study was observed. One patient with unknown genotypes was classified as mild Scheie form and one as sever hurler form. Examining the *IDUA* gene promoter and a powerful method to detect the duplications could indicate the molecular basis of the disease in these cases. Moreover, modifying factors may highly effect on metabolic phenotype.^21^, ^22^

In short, in this study, we extended information on the mutational spectrum of the *IDUA* gene by reporting data representative of the Iranian MPSI population. A spectrum of five mutations and a total of 32 different genotypes were identified. The detection rate was 90.48%. Our findings should be used as an effective tool for all clinical and diagnostic workplaces in Iran, and they also provide useful information for more clinical trials. In addition, results herein will help to complete relevant genetic information on the Middle East populations and provide an acceptable contribution to the large‐scale Asian and Middle East studies.

## CONFLICT OF INTEREST

Dr M Taghikhani has received research grants from Tarbiat Modares University. Dr Sh Khatami declares no potential conflicts of interest with respect to the research, authorship, and/or publication of this article. Dr MS Hakhamaneshi declares that he has no conflict of interest. Dr M Abdi declares no potential conflicts of interest with respect to the research, authorship, and/or publication of this article. Dr MR Alaei declares that he has no conflict of interest. Dr D Zamanfar declares that he has no conflict of interest. Dr R Vakili declares that he has no conflict of interest.

## AUTHORS' CONTRIBUTIONS

All authors contributed equally in this work.

## ETHICAL APPROVAL

All procedures performed in studies involving human participants were in accordance with the ethical standards of the ethics committee of Tarbiat Modares University and Pasteur Institute of Iran and with the 1964 Helsinki declaration and its later amendments or comparable ethical standards.

## INFORMED CONSENT

Informed consent was obtained from all individual participants included in the study.

## FINANCIAL DISCLOSURE

The author has no financial relationships relevant to this article to disclose.
